# Occurrence of Myocarditis in Patients Immunized with Different Types of COVID-19 Vaccines: A Systematic Review and Meta-Analysis

**DOI:** 10.1016/j.virusres.2026.199748

**Published:** 2026-05-20

**Authors:** Juan Jose Valenzuela-Fuenzalida, Laura Moyano Valarezo, Vicente Silva, Fernanda Delgado, Diego Nazar-Izquierdo, Alejandro Bruna-Mejias, Pablo Nova-Baeza, Mathias Orellana Donoso, Gustavo Oyanedel- Amaro, Gloria Cifuentes-Suazo, Maria P Moya, Juan Sanchis- Gimeno, Marko Konschake, Jessica Paola Loaiza-Giraldo

**Affiliations:** a Departamento de Morfología, Facultad de Medicina, Universidad Andrés Bello, Santiago 8370146, Chile; b Departamento de Ciencias Química y Biológicas, Facultad de Ciencias de la Salud, Universidad Bernardo O’Higgins, Santiago 8370993, Chile; c Facultad de Medicina y Ciencia, Universidad San Sebastián, Lota 2465, Santiago 7510157, Chile; d Escuela de Medicina, Universidad Finis Terrae, Santiago 7501015, Chile; e Faculty of Health and Social Sciences, Universidad de Las Américas, 8370040, Santiago, Chile; f Facultad de Medicina, Carrera de Odontología, Universidad Católica de la Santísima Concepción, Av. Alonso de Ribera 2850, Concepción 4090541, Chile; g Facultad de Ciencias de la Salud, Universidad Autónoma de Chile, Santiago 7500912, Chile; h GIAVAL Research Group, Department of Anatomy and Human Embryology, Faculty of Medicine, University of Valencia, 46001 Valencia, Spain; i Institute of Clinical and Functional Anatomy, Medical University of Innsbruck (MUI), Müllerstr. 59, 6020 Innsbruck, Austria; j Facultad de Ciencias de la Salud, Universidad Central del Valle del Cauca (UCEVA), Tuluá 763022, Colombia

**Keywords:** COVID-19 Vaccines, Myocarditis, mRNA Vaccines, Adenoviral Vector Vaccines, Vaccine Safety, Adverse Events, Pharmacovigilance, Systematic Review, Meta-Analysis

## Abstract

**Background:**

Myocarditis has emerged as a rare but clinically relevant adverse event reported after COVID-19 vaccination, particularly following widespread use of vaccines based on novel molecular platforms. Given variability in vaccine technologies, population characteristics, and surveillance methodologies, a comprehensive quantitative synthesis is required to better characterize the occurrence of post-vaccination myocarditis. This study aimed to characterize the distribution of reported myocarditis cases among individuals receiving COVID-19 vaccines, including mRNA, viral vector, and protein-subunit platforms, and to synthesize available evidence on reported post-vaccination myocarditis across different demographic and geographic subgroups.

**Methods:**

This systematic review and meta-analysis was conducted in accordance with PRISMA guidelines and registered in PROSPERO (CRD420251118332). MEDLINE, Web of Science, Scopus, CINAHL, Google Scholar, and LILACS were searched from inception to January 2024 for observational studies reporting myocarditis following COVID-19 vaccination. Cohort, case-control, cross-sectional studies, and case series were eligible. Study quality was assessed using the ROBINS-I tool. Random-effects models were applied to estimate pooled proportions with 95% confidence intervals (CIs). Statistical heterogeneity was quantified using the I² statistic, and prespecified subgroup analyses were performed by sex, age, geographic region, and vaccine platform. Publication bias was explored using funnel plot analysis.

**Results:**

Fifty-nine studies comprising 196,478,861 vaccinated individuals and 13,348 reported myocarditis cases were included. Due to substantial heterogeneity in study designs and denominators, pooled estimates represent the proportion of myocarditis cases within reported samples rather than population-level incidence or risk. Across all included studies, the pooled proportion of myocarditis cases within reported study samples was 34% (95% CI: 19–50%), with considerable heterogeneity (I² = 100%). These estimates should not be interpreted as population-level incidence or risk. Reported myocarditis cases were more frequently observed among males (72%, 95% CI: 58–86%) than females (56%, 95% CI: 35–77%) and were predominantly identified in individuals younger than 40 years. Subgroup analyses by region and vaccine platform should be interpreted cautiously due to methodological variability and potential selection bias. Funnel plot asymmetry suggested possible small-study effects.

**Conclusions:**

This systematic review and meta-analysis aimed to characterize the distribution of reported myocarditis cases following COVID-19 vaccination rather than to estimate population-level incidence or risk. Although pooled proportions within reported samples were substantial, these estimates do not reflect population-level incidence. Available evidence suggests that myocarditis following COVID-19 vaccination remains uncommon at the population level, predominantly affecting younger males and more commonly reported after mRNA-based vaccines. Most reported cases appear to follow a benign and self-limited clinical course. These findings support the overall favorable benefit–risk profile of COVID-19 vaccines while underscoring the need for continued pharmacovigilance and more robust epidemiological studies to better characterize the epidemiology of vaccine-associated myocarditis.

## Highlights

• Myocarditis cases were analyzed as descriptive proportions, not incidence.
• Higher proportions were observed in younger individuals, particularly males.
• mRNA vaccines accounted for a larger share of reported cases.
• Substantial heterogeneity limits comparability of pooled estimates.

## 1 Introduction

Coronavirus disease 2019 (COVID-19), caused by the SARS-CoV-2 virus, has represented one of the most significant public health challenges of the 21st century. Since its first detection on the Asian continent, the virus rapidly spread worldwide, leading to a pandemic that profoundly affected global health systems and the lives of millions of individuals. While most people infected with SARS-CoV-2 experience mild to moderate respiratory illness and recover spontaneously, a substantial proportion develop severe manifestations requiring hospitalization and intensive care (Hoang, 2021 Mar 24). Typical symptoms of COVID-19, which generally appear 2 to 14 days after exposure, include dry cough, dyspnea, ageusia, anosmia, and profound fatigue (Khalili et al., 2020 Jun 29).

Beyond its respiratory involvement, SARS-CoV-2 has demonstrated the capacity to affect multiple organ systems, including the cardiovascular system. Cardiac involvement may arise through direct viral invasion of myocardial cells or indirect mechanisms such as systemic inflammation and thrombosis, leading to complications including myocarditis, arrhythmias, and heart failure (Hausvater et al., 2020 Sep). In the context of this global health emergency, the accelerated development of effective vaccines was crucial. Multiple vaccines were produced in less than 11 months following the genetic sequencing of SARS-CoV-2, demonstrating high efficacy in reducing severe disease, hospitalizations, and mortality (Khalili et al., 2020 Jun 29).

Approved COVID-19 vaccines differ substantially in their molecular platforms, a factor that may contribute to variation in adverse event profiles. mRNA-based vaccines include Pfizer-BioNTech (BNT162b2) and Moderna (mRNA-1273); viral vector vaccines comprise AstraZeneca (ChAdOx1 nCoV-19), Johnson & Johnson (Ad26.COV2.S), and Sputnik V; and protein subunit vaccines include Novavax (NVX-CoV2373) and Zifivax (ZF2001). Understanding platform-specific differences is essential when evaluating rare but clinically relevant adverse outcomes. (Xu et al., 2023 Mar 3)

**Figure 1:**
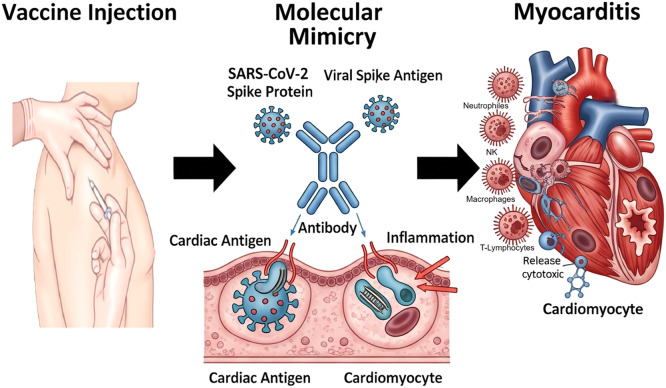
Hypothesized molecular mimicry–mediated mechanism of myocarditis following COVID-19 vaccination.

This review focuses on myocarditis as a potential adverse event following COVID-19 immunization. Myocarditis, defined as inflammation of the myocardium, can compromise the heart’s ability to effectively pump blood, resulting in reduced ventricular function and, in severe cases, life-threatening heart failure (6). Its etiologies are diverse and often difficult to determine at presentation. Common causes include viral infections (e.g., enterovirus, adenovirus, parvovirus B19, and SARS-CoV-2), drug reactions, toxins, and autoimmune or systemic inflammatory disorders. Etiologic identification is challenging because myocarditis is a dynamic process with heterogeneous clinical expressions (Figure 1) (Rostami et al., 2021 Mar).

This schematic illustrates a proposed immune-mediated mechanism underlying myocarditis following COVID-19 vaccination. After vaccine administration, antibodies are generated against the SARS-CoV-2 spike protein. Due to structural similarities between viral spike antigens and certain cardiac antigens (molecular mimicry), these antibodies may cross-react with cardiomyocyte-associated proteins. This cross-reactivity can trigger an inflammatory cascade characterized by immune cell recruitment (including macrophages, neutrophils, natural killer cells, and T lymphocytes), release of cytotoxic mediators, and subsequent myocardial inflammation, potentially resulting in myocarditis.

Clinical presentations range from asymptomatic or mild forms to fulminant myocarditis with cardiogenic shock. Symptoms may include precordial pain (anginal or pleuritic), fatigue, peripheral edema, arrhythmias from supraventricular tachycardias to malignant ventricular arrhythmias exertional or resting dyspnea, syncope, and nonspecific viral-like prodromal symptoms such as fever, headache, odynophagia, myalgias, and arthralgias. Due to overlap with other acute cardiac conditions, such as acute myocardial infarction, the onset of unexplained chest pain or dyspnea warrants urgent medical evaluation (Rostami et al., 2021 Mar). Diagnosis of myocarditis requires a multimodal approach incorporating clinical assessment, cardiac biomarkers, electrocardiography, and echocardiography. Cardiac magnetic resonance imaging provides additional diagnostic clarity, whereas endomyocardial biopsy remains the gold standard but is reserved for selected cases due to its invasive nature and associated risks (Carmona et al., 2023 Nov).

Despite the overall success and favorable safety profile of COVID-19 vaccines, post-authorization surveillance systems have identified rare but clinically significant adverse events, including myocarditis and pericarditis. The exceptionally rapid development timeline of COVID-19 vaccines meant that long-term cardiovascular safety data were limited at the time of emergency authorization and mass distribution (Xu et al., 2023 Mar 3). Reported post-vaccination myocarditis and pericarditis appear more frequently among hospitalized patients and individuals with preexisting cardiac conditions. These events exhibit a notable male predominance, especially in males over 50 years of age and in pediatric populations, although their true incidence and prevalence remain incompletely defined (Monda et al., 2024 Jun).

While the prevalence of myocarditis after vaccination is low, it remains clinically relevant. Importantly, the risk of myocarditis following SARS-CoV-2 infection is significantly higher than the risk associated with vaccination (Ammirati and Moslehi, 2023 Apr 4). Proposed mechanisms for vaccine-related adverse events include direct reactions to vaccine components, quality issues during the immunization process, or errors in handling and administration. Idiosyncratic reactions also play a role, arising from individualized immune responses whose underlying mechanisms are not fully understood (Bozkurt et al., 2021 Aug 10). For post-vaccination myocarditis specifically, several theories have been proposed. One posits that antibodies against the SARS-CoV-2 spike protein cross-react with cardiac antigens via molecular mimicry. Another suggests that mRNA vaccines activate innate immune responses through RNA-sensing receptors, thereby triggering inflammatory pathways that could lead to myocarditis (Chimenti et al., 2022 Mar, Abou Hassan et al., 2021 Aug 3).

Recent large-scale observational studies have investigated vaccine-related adverse events affecting multiple organ systems using robust epidemiological designs such as matched cohort studies and self-controlled case series. For example, studies evaluating uveitis risk and neuro-ophthalmic adverse events following COVID-19 vaccination have demonstrated the value of these methodologies in estimating temporal associations and relative risk in real-world populations. These approaches provide important methodological context for interpreting vaccine safety signals and highlight the complexity of assessing rare adverse events across heterogeneous data sources.

Unlike previous systematic reviews focused primarily on incidence estimates or clinical outcomes, the present study aimed to provide a descriptive synthesis of reported myocarditis cases across different vaccine platforms, demographic subgroups, and geographic regions while explicitly addressing the methodological heterogeneity of available evidence.

### 1.1 Objective

To characterize the distribution of reported myocarditis cases following immunization with different COVID-19 vaccine platforms (mRNA, viral vector, and protein subunit vaccines) and to synthesize available evidence through a systematic review and meta-analysis.

## 2 Methods

### 2.1 Protocol and Registration

This systematic review and meta-analysis were conducted and reported in accordance with the Preferred Reporting Items for Systematic Reviews and Meta-Analyses (PRISMA) guidelines (Page et al., 2021 Mar 29). The study protocol was prospectively registered in the International Prospective Register of Systematic Reviews (PROSPERO) under the registration number **CRD420251118332**.

### 2.2 Eligibility Criteria

Studies were included based on the following criteria: (1) Population: studies reporting patients who developed myocarditis or pericarditis following immunization with any type of COVID-19 vaccine; (2) Outcomes: studies describing the occurrence, clinical presentation, diagnostic findings, or temporal association of myocarditis after vaccination; (3) Study design: original research articles, including observational studies (cohort, case-control, and cross-sectional) and case series published in peer-reviewed journals in English or Spanish (Figure Supplementary 1). Due to the exploratory nature of this review and the limited availability of large-scale comparative studies for certain subgroups, different observational study designs were included. These designs differ in terms of case ascertainment, denominators, and susceptibility to bias, which was taken into account when interpreting pooled estimates. Exclusion criteria were: (1) Population: animal studies; (2) Outcome: studies evaluating myocarditis unrelated to COVID-19 vaccination; (3) Study design: reviews, editorials, letters without extractable data, and conference abstracts that lacked sufficient methodological information. The ROBINS-I tool was selected to evaluate risk of bias in non-randomized studies; however, given the inclusion of diverse observational designs, including descriptive studies and case series, its applicability may be limited in certain contexts. Therefore, risk of bias assessments should be interpreted with caution, particularly for studies lacking a defined comparison group.

### 2.3 Electronic Search

A comprehensive literature search was conducted in MEDLINE (via PubMed), Web of Science, Google Scholar, CINAHL, Scopus, and LILACS from inception to January 2024. The search strategy combined controlled vocabulary and free-text terms, including “Myocarditis,” “Pericarditis,” “COVID-19 Vaccines,” “mRNA vaccines,” “Viral vector vaccines,” and “Protein-subunit vaccines,” using the Boolean operators **AND, OR**, and **NOT**. Based on a PICO framework, we considered: **Population:** patients receiving any COVID-19 vaccine; **Intervention/Exposure:** immunization with mRNA, viral vector, or protein-subunit vaccines; **Comparison:** unvaccinated populations or other vaccine platforms (when available); **Outcomes:** incidence or diagnosis of myocarditis. Detailed search strategies for each database are provided in the Supplementary Material (Supplementary Table S1).

### 2.4 Study Selection

Two reviewers (LM and VS) independently and in duplicate screened all titles and abstracts yielded by the search. Full-text manuscripts were obtained for all studies meeting the inclusion criteria or when eligibility could not be determined from the abstract. Any disagreements were resolved by consensus, and unresolved discrepancies were adjudicated by a third reviewer (JJV-F). Additional studies identified through other methods included manual screening of reference lists from eligible articles and relevant review papers.

### 2.5 Data Collection Process

Two authors **(M-D and JJV-F) i**ndependently extracted relevant data using a standardized extraction form. Extracted variables included: (I) authors and publication year; (II) study design and sample size; (III) incidence and clinical characteristics of myocarditis; (IV) type of COVID-19 vaccine administered; (V) geographic region; (VI) age; (VII) sex; and (VIII) diagnostic criteria and treatment. Any disagreement in data extraction was resolved by consensus.

### 2.6 Assessment of Methodological Quality

The methodological quality and risk of bias for each included study were assessed using the Risk of Bias in Non-Randomized Studies of Interventions (ROBINS-I) tool (Li et al., 2025 Oct). This tool evaluates potential bias across the following domains: confounding, participant selection, classification of interventions, deviations from intended interventions, missing data, outcome measurement, and selective reporting.

Each domain was rated as low, moderate, serious, or critical risk of bias. A low risk indicates that a study is comparable to a well-conducted randomized trial in that specific domain, while a moderate risk reflects acceptable quality for a non-randomized design. Serious or critical risk indicates major methodological limitations. When insufficient information was available for a domain, it was rated as “no information.” The overall risk of bias for each study corresponded to the highest risk rating across all domains (Pace-Loscos et al., 2024 Jun 12).

### 2.7 Statistical Analysis

Random-effects meta-analyses were performed to estimate pooled proportions with corresponding 95% confidence intervals (CIs), accounting for between-study variability. Proportions were transformed using appropriate variance-stabilizing methods when necessary. Statistical heterogeneity was assessed using the I² statistic, with values >75% indicating substantial heterogeneity. Prespecified subgroup analyses were conducted according to sex, age group, geographic region, and vaccine platform. Due to the expected clinical and methodological heterogeneity across studies, subgroup findings were interpreted descriptively. Publication bias and small-study effects were explored through visual inspection of funnel plots. Given the inclusion of heterogeneous study designs, including case series and large population-based cohort studies with markedly different denominators and ascertainment methods, pooled estimates were interpreted as proportions within reported samples rather than true measures of incidence or prevalence. The combination of fundamentally different study designs may lead to distorted effect estimates and limits the epidemiological interpretability of the findings. Therefore, these results do not represent population-level risk and cannot be interpreted as estimates of myocarditis incidence following vaccination. All statistical analyses were performed using R software (version 4.3.0; R Foundation for Statistical Computing, Vienna, Austria) using the ‘meta’ and ‘metafor’ packages.

## 3 Results

**Figure 2:**
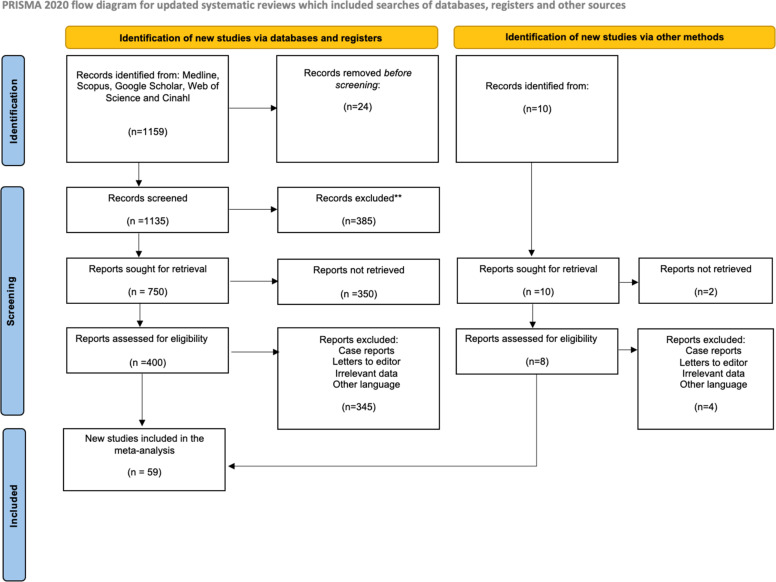
Search diagram for this review.

For the descriptive analysis of reported myocarditis cases following COVID-19 vaccination, 59 studies were included (Table 1) (Yap et al., 2022 Feb, Shaw et al., 2021 Sep, Stowe et al., 2023 Jun 7, Yonker et al., 2023 Mar 14, Goddard et al., 2022 Aug 19, Asaduzzaman et al., 2022 Jul 15, Bouchaala et al., 2023 Apr 20, Ilonze and Guglin, 2022 Nov, Saadi et al., 2022 Nov, Olagunju et al., 2022 Jan-Dec, D'Angelo et al., 2021 Oct, Chow and Lai, 2022 Aug 4, Murakami et al., 2022 Feb 15, Kang et al., 2022 Mar 20, Loch et al., 2023 Jun, Patrignani et al., 2021 Nov, Hudson et al., 2021 Jul 26, Pasha et al., 2022 Jul 7, Kerneis et al., 2021 Jun-Jul, Patel et al., 2022 May 3, Patone et al., 2022 Sep 6, Patone et al., 2022 Feb, Schwab et al., 2023 Mar, Di Dedda et al., 2022 Jul, Puchalski et al., 2022 Mar 15, Shiyovich et al., 2022 Jul 21, Chen et al., 2024 May 30, Nakahara et al., 2023 Sep, Koizumi et al., 2021 Dec 20, Le Vu et al., 2024 Sep 5, Cho et al., 2023 Jun 25, Abu Mouch et al., 2021 Jun 29, Patel et al., 2021 Sep 9, Starekova et al., 2021 Nov, Nguyen et al., 2021 Dec, Lin et al., 2022 Sep, Vila-Olives et al., 2024 May, Truong et al., 2022 Feb, Ahn et al., 2024 Dec 23, Banala et al., 2023 Feb, Chelala et al., 2022 Apr, Foltran et al., 2022 Mar 2, Fronza et al., 2022 Sep, Hassanzadeh et al., 2022 Apr 15, Hause et al., 2022 Aug 1, Husby et al., 2021 Dec 16, Jain et al., 2021 Nov, King et al., 2021 Aug, Mörz, 2022 Oct 1, Ng et al., 2023 Dec 7, Witberg et al., 2021 Dec 2, Singh et al., 2021 Jun 14, Vago et al., 2022 Sep 15, Rafaniello et al., 2022 Apr 25, Ferchichi et al., 2022 Dec, Petersen et al., 2023 Apr, Hajjo et al., 2021 Oct 15, Montgomery et al., 2023 Jan, Moosmann et al., 2022 Apr 14). These studies comprised a total of 196,478,861 vaccinated individuals and 13,348 reported cases. A total of 17 forest plots were generated and are presented in the following sections (Figure 2).

**Table 1:** Characteristics of all included studies reporting myocarditis following COVID-19 vaccination (n = 59).

Author (Year)	Sample size	Age and sex	Myocarditis cases	Vaccine type	Dose number	Other reported adverse events	Previous conditions / comorbidities
Yap (2022)	25 cases from 7,183,889 administered doses	80% male; median age 23 years (range 12-55)	25 (12 confirmed, 13 probable); 11 with concomitant pericarditis	mRNA (BNT162b2, mRNA-1273)	First dose (9); second dose (16)	Pericarditis; systemic symptoms	Not reported
Shaw (2021)	4	Males 16, 24; Females 17, 31 years	4	BNT162b2 (3); mRNA-1273 (1)	First (2); second (2)	Systemic symptoms	Prior SARS-CoV-2 infection (2 cases)
Stowe (2023)	51,385,436	Wide age range; both sexes	3,756	ChAdOx1; BNT162b2; mRNA-1273	Not specified	Pericarditis	Prior SARS-CoV-2 infection (297 hospitalized cases)
Yonker (2023)	61	Mean age 16 years; 13 M / 3 F	16	mRNA vaccines	First (2); second (12); third (2)	Systemic symptoms	Not reported
Goddard (2022)	79	100% male; median age 22.8 years	18	BNT162b2; mRNA-1273	First and second doses	Pericarditis	Not reported
Asaduzzaman (2022)	1	Female, 15 years	1	BNT162b2	Second dose	Encephalopathy; thrombocytopenia; seizures	None reported
Bouchaala (2023)	2	Males, 26 and 46 years	2	ChAdOx1	Second dose	Not reported	None
Ilonze (2022)	238	Mean age 27.4 ± 16 years; 208 M / 30 F	238	BNT162b2; mRNA-1273; ChAdOx1; Janssen; Sinovac	First (56); second (182)	Cardiogenic shock; death	Prior COVID-19 infection (7 cases)
Saadi (2022)	1	Male, 18 years	1	mRNA-1273	Third dose	Not reported	None
Olangaju (2021)	1	Male, 19 years	1	mRNA-1273	First dose	Not reported	Asthma; marijuana use
D’Angelo (2022)	1	Male, 30 years	1	BNT162b2	Second dose	Chest pain; dyspnea	None
Chow (2022)	1	Female, 45 years	1	mRNA-1273	First dose	Syncope	None
Murakami (2022)	2	Males, 27 and 37 years	2	BNT162b2	First (1); second (1)	Not reported	None
Kang (2021)	1	Female, 48 years	1	ChAdOx1 followed by BNT162b2	Mixed	Heart transplant; death	Hypothyroidism
Loch (2023)	1	Male, 34 years	1	BNT162b2	Second dose	Ventricular dysfunction; thrombus	Asthma; former smoker
Patrignani (2021)	1	Male, 56 years	1	BNT162b2	First dose	Hypotension; tachycardia	Not reported
Hudson (2021)	2	Males, 22-24 years	2	BNT162b2	Second dose	Fever; chest pain	Not reported
Pasha (2022)	1	Male, 45 years	1	mRNA-1273	Second dose	Chest pain	Prior SARS-CoV-2 infection
Kerneis (2022)	214	Mean age 35 years; 131 M / 83 F	85	BNT162b2; mRNA-1273	Not reported	Pericarditis (22%)	None reported
Patel (2022)	201	Mean age 15.7 years	9 vaccine-related	mRNA vaccines	Second dose	Lymphopenia; thrombocytopenia	Asthma; obesity
Patone (2022)	38,897,774	Adults; both sexes	2,861	ChAdOx1; BNT162b2; mRNA-1273	First-third doses	Not reported	Not reported
Patone b (2022)	38,615,491	Adults; both sexes	1,615	ChAdOx1; BNT162b2; mRNA-1273	Second dose	Pericarditis; arrhythmias	Not reported
Schwab (2022)	25	Mean age 58 years	5	BNT162b2; mRNA-1273	Mostly first dose	Nausea	COPD; diabetes; hypertension
Di Dedda (2023)	27	Mean age 36.6 years	23	Vaxzevria; BNT162b2; mRNA-1273	First (12); second (15)	Chest pain; dyspnea	Hypertension; diabetes; smoking
Puchalski (2022)	5	Males, 15-17 years	5	BNT162b2	First (3); second (2)	Chest pain; fever	Obesity
Shiyovich (2022)	15	100% male; mean age 32 years	15	BNT162b2	First (5); second (10)	Fever; chest pain	Hypertension; dyslipidemia
Chen (2024)	622,447	Adults; both sexes	2,857	mRNA vaccines	Not reported	Not reported	Not reported
Nakahara (2023)	1,003	Mean age 56.8 years (vaccinated)	Not specified	mRNA vaccines	Not reported	Not reported	Malignancy; hypertension
Koizumi (2023)	2	Males, 22 and 27 years	2	mRNA-1273	Second dose	Not reported	Not reported
Vu (2024)	7,911	Mean age 34 years; both sexes	7,911	BNT162b2; mRNA-1273	Second dose	Not reported	Prior myocarditis; SARS-CoV-2 infection
Cho (2023)	44,276,704	Adults; both sexes	480	BNT162b2; mRNA-1273; ChAdOx1	Third dose	Chest pain; dyspnea	Cardiovascular disease; diabetes
Abu (2021)	6	Mean age 25 years; all male	6	BNT162b2	First–second dose	Not reported	Not reported
Patel (2021)	5	Males, 19-37 years	5	BNT162b2; mRNA-1273	First (1); second (4)	Pericarditis	Asthma
Starekova (2021)	5	Mean age 25 years	5	BNT162b2; mRNA-1273	Second dose	Fever; chest pain	Not reported
Nguyen (2021)	1	Male, 20 years	1	mRNA-1273	First dose	Fever; chest pain	Smoker
Lin (2022)	1	Male, 26 years	1	BNT162b2	Second dose	Epigastric discomfort	Not reported
Vila (2024)	2	Males, 22 and 46 years	2	BNT162b2	First and second dose	Cardiac arrest; syncope	Not reported
Truong (2022)	139	Median age 15.8 years	49 confirmed	Not reported	Not reported	Not reported	Prior SARS-CoV-2 infection
Ahn (2024)	3,709,063	Adolescents	115 myocarditis	BNT162b2	First–third doses	Fever; syncope	Not reported
Banala (2023)	1	Male, 16 years	1	BNT162b2	Second dose	Chest pain; fever	Not reported
Chelala (2022)	52	Mean age 17.2 years; males	5	BNT162b2; mRNA-1273	Second dose	Chest pain	Marijuana use
Foltran (2022)	4,942	Mean age 15.6 years	191 myocarditis	BNT162b2; mRNA-1273	Mixed	Chest pain; dyspnea	Not reported
Fronza (2022)	92	Mean age 31 years	21	BNT162b2; mRNA-1273	Mostly second dose	Palpitations; dyspnea	Smoking
Hassanzadeh (2022)	1	Female, 32 years	1	ChAdOx1	First dose	Tachycardia; dyspnea	Not reported
Hause (2022)	48,795	Children 5-11 years	15	BNT162b2	First and second doses	Fever; headache	Asthma; diabetes
Husby (2021)	4,931,775	≥12 years	269	BNT162b2; mRNA-1273	First and second doses	Not reported	Asthma; diabetes
Jain (2021)	63	Mean age 15.6 years	63	BNT162b2; mRNA-1273	Mostly second dose	Fever; chest pain	Not reported
King (2021)	4	Ages 20-30 years	4	BNT162b2; mRNA-1273	Second dose	Chest pain	Not reported
Morz (2022)	1	Male, 73 years	1	ChAdOx1; BNT162b2	Third dose	Cardiovascular symptoms	Parkinson’s disease
Ng (2023)	67	Mean age 30 years	0	BNT162b2; Sinovac	First and second doses	Chest pain; nausea	Hypertension
Witberg (2021)	2,558,421	Mean age 44 years	54	BNT162b2	First and second doses	Palpitations; dyspnea	Hypertension; dyslipidemia
Singh (2021)	1	Male, 24 years	1	BNT162b2	Second dose	Fever; fatigue	Smoker
Vago (2022)	16	Mean age 22 ± 7 years	16	BNT162b2; mRNA-1273; Sputnik V	First–third doses	Not reported	Prior SARS-CoV-2 infection
Rafaniello (2022)	778	Adolescents and adults	778	mRNA vaccines	Mostly second dose	Not reported	Not reported
Ferchichi (2022)	5	Mean age 30 years	5	BNT162b2; mRNA-1273; Vaxzevria	First and second doses	Chest pain; dyspnea	None
Petersen (2023)	4	Adults	4 suspected	mRNA vaccines	Not reported	Not reported	Not reported
Hajjo (2021)	2,642	Adults	1,579 myocarditis	BNT162b2; mRNA-1273; Janssen	All doses	Not reported	Not reported
Montgomery (2023)	179	Mean age 39 years	3 myocarditis	BNT162b2; mRNA-1273; Janssen	Various	Chest pain; dyspnea	Not reported
Moosmann (2022)	2	Mean age 13 years	2	BNT162b2	First and second doses	Chest pain; myocardial edema	Not reported

### 3.1 Distribution of Reported Cases

**Figure 3:**
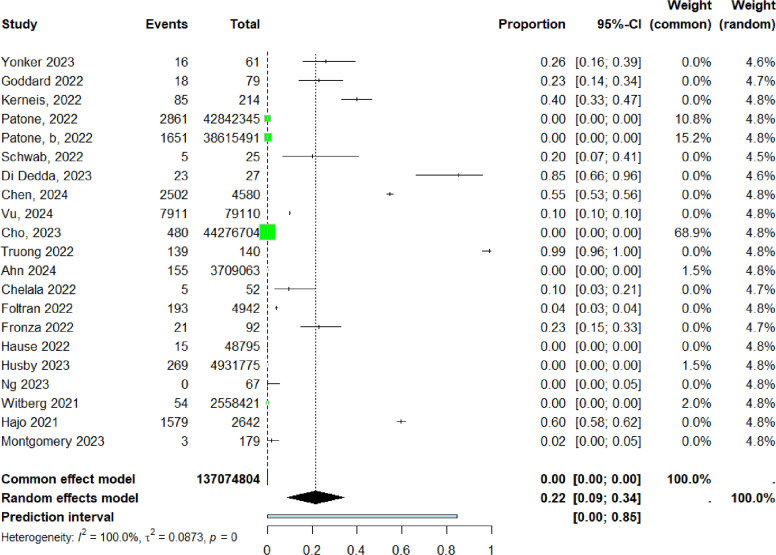
Forest plot prevalence all studies.

**Figure 4:**
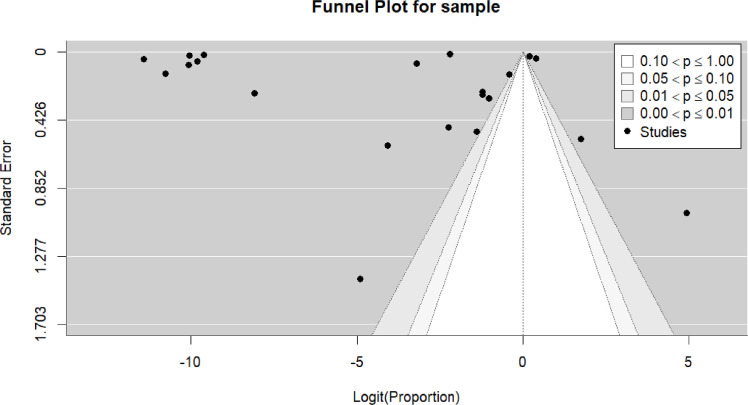
Funnel plot of all included studies.

Twenty-one studies (Yap et al., 2022 Feb, Yonker et al., 2023 Mar 14, Goddard et al., 2022 Aug 19, Ilonze and Guglin, 2022 Nov, Kerneis et al., 2021 Jun-Jul, Patel et al., 2022 May 3, Patone et al., 2022 Sep 6, Patone et al., 2022 Feb, Schwab et al., 2023 Mar, Di Dedda et al., 2022 Jul, Chen et al., 2024 May 30, Le Vu et al., 2024 Sep 5, Cho et al., 2023 Jun 25, Truong et al., 2022 Feb, Ahn et al., 2024 Dec 23, Chelala et al., 2022 Apr, Foltran et al., 2022 Mar 2, Fronza et al., 2022 Sep, Hause et al., 2022 Aug 1, Husby et al., 2021 Dec 16, Jain et al., 2021 Nov, Ng et al., 2023 Dec 7, Witberg et al., 2021 Dec 2, Hajjo et al., 2021 Oct 15, Montgomery et al., 2023 Jan) were included in the pooled analysis of reported myocarditis cases following vaccination. The pooled proportion of reported cases was 22% (95% CI: 9%–34%). Heterogeneity remained high (I² = 100%) (Figure 3). Funnel plot inspection suggested asymmetry, indicating potential publication bias and/or small-study effects (Figure 4 and Table 2).

**Table 2:** Population-based studies evaluating myocarditis following COVID-19 vaccination.

Author (Year)	Country / Data source	Sample size	Age (years)	Sex	Myocarditis cases	Vaccine type	Dose associated	Key findings
**Stowe (2023)**	National surveillance database	51,385,436	≥12 (age-stratified)	M/F	3,756	ChAdOx1, BNT162b2, mRNA-1273	1st-2nd	Higher risk after mRNA vaccines, particularly in younger males; concomitant pericarditis reported
**Patone (2022)**	National health records	38,897,774	Adults	M/F	2,861	ChAdOx1, BNT162b2, mRNA-1273	1st-3rd	Myocarditis mainly after first or second dose within 1-28 days
**Patone (2022b)**	National health records	38,615,491	Adults	M/F	1,615	ChAdOx1, BNT162b2, mRNA-1273	2nd	Myocarditis and/or death within 28 days; pericarditis and arrhythmias also reported
**Witberg (2021)**	National vaccination program	2,558,421	Mean 44	M/F	54	BNT162b2	1st-2nd	Higher incidence after second dose, predominantly in young males
**Husby (2021)**	Nationwide cohort	4,931,775	≥12	M/F	269	BNT162b2, mRNA-1273	1st-2nd	Increased risk in males aged 12-39 years
**Hause (2022)**	Passive surveillance (children)	48,795	5–11	M/F	15	BNT162b2	1st-2nd	Rare myocarditis in pediatric population
**Cho (2023)**	Nationwide database	44,276,704	Mean 45	M/F	480	BNT162b2, mRNA-1273, ChAdOx1, Ad26	3rd	Low absolute risk; mostly mild clinical course
**Chen (2024)**	National vaccination registry	622,447	Adults	M/F	2,857	Pfizer-BioNTech, Moderna (original/bivalent)	Not specified	Myocarditis more frequent after original mRNA formulations
**Ahn (2024)**	Nationwide adolescent cohort	3,709,063	Mean 17	M/F	115 myocarditis	BNT162b2	1st-3rd	Majority occurred after second dose; myopericarditis and pericarditis also observed
**Foltran (2022)**	Population registry (adolescents)	4,942	Mean 15.6	M/F	191 myocarditis	BNT162b2, mRNA-1273	1st-2nd	Predominance after second dose; chest pain most common symptom
**Vu (2024)**	Large observational cohort	7,911	Mean 34	M/F	7,911*	BNT162b2, mRNA-1273	2nd	Prior myocarditis and SARS-CoV-2 infection increased risk
**Rafaniello (2022)**	Pharmacovigilance database	Not reported	Adolescents–elderly	M/F	778	mRNA vaccines	1st-2nd	Higher reporting rate after second dose
**Hajjo (2021)**	Global pharmacovigilance	2,642	Not reported	M/F	1,579 myocarditis	Pfizer, Moderna, Janssen	1st-3rd	Myocarditis more frequently reported than pericarditis
**Montgomery (2023)**	Military health system	179	Mean 39	M/F	3 myocarditis	Pfizer, Moderna, Janssen	1st-booster	Rare events with favorable outcomes
**Nakahara (2023)**	Hospital-based cohort	1,003	Mean ∼55	M/F	Not specified	Various	Not specified	Higher comorbidity burden in vaccinated myocarditis cases

Data are presented as reported in the original studies.

**Abbreviations:** BNT162b2, Pfizer–BioNTech COVID-19 vaccine; mRNA-1273, Moderna COVID-19 vaccine; Ad26, adenoviral vector vaccine.

### 3.2 Distribution of Cases by Sex (Female)

**Figure 5:**
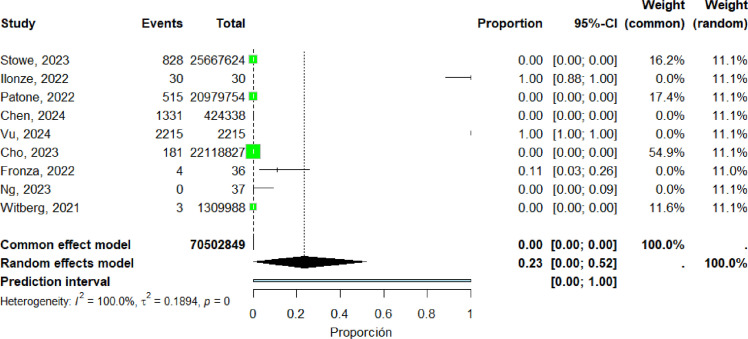
forest plot female included studies.

Nine studies (Stowe et al., 2023 Jun 7, Ilonze and Guglin, 2022 Nov, Patone et al., 2022 Sep 6, Chen et al., 2024 May 30, Le Vu et al., 2024 Sep 5, Cho et al., 2023 Jun 25, Fronza et al., 2022 Sep, Ng et al., 2023 Dec 7, Witberg et al., 2021 Dec 2) were included in the pooled analysis of reported myocarditis cases among women with a sample size greater than 20 participants. The pooled proportion of reported cases was 23% (95% CI: 0%–52%). Heterogeneity remained high (I² = 100%) (Figure 5).

### 3.3 Distribution of Cases in Men

**Figure 6:**
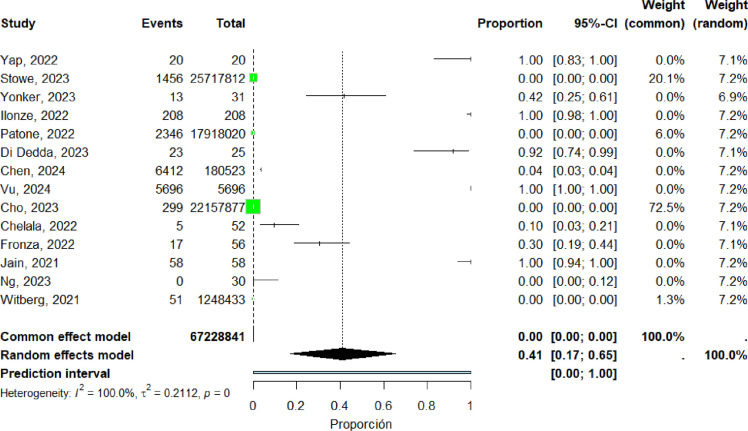
forest plot male includes studies.

Forty-one studies (Yap et al., 2022 Feb, Shaw et al., 2021 Sep, Stowe et al., 2023 Jun 7, Yonker et al., 2023 Mar 14, Goddard et al., 2022 Aug 19, Ilonze and Guglin, 2022 Nov, Saadi et al., 2022 Nov, Olagunju et al., 2022 Jan-Dec, D'Angelo et al., 2021 Oct, Murakami et al., 2022 Feb 15, Loch et al., 2023 Jun, Patrignani et al., 2021 Nov, Hudson et al., 2021 Jul 26, Pasha et al., 2022 Jul 7, Patone et al., 2022 Sep 6, Di Dedda et al., 2022 Jul, Puchalski et al., 2022 Mar 15, Shiyovich et al., 2022 Jul 21, Chen et al., 2024 May 30, Koizumi et al., 2021 Dec 20, Le Vu et al., 2024 Sep 5, Cho et al., 2023 Jun 25, Abu Mouch et al., 2021 Jun 29, Patel et al., 2021 Sep 9, Starekova et al., 2021 Nov, Nguyen et al., 2021 Dec, Lin et al., 2022 Sep, Vila-Olives et al., 2024 May, Banala et al., 2023 Feb, Chelala et al., 2022 Apr, Fronza et al., 2022 Sep, Jain et al., 2021 Nov, King et al., 2021 Aug, Mörz, 2022 Oct 1, Ng et al., 2023 Dec 7, Witberg et al., 2021 Dec 2, Singh et al., 2021 Jun 14, Vago et al., 2022 Sep 15, Ferchichi et al., 2022 Dec, Petersen et al., 2023 Apr, Moosmann et al., 2022 Apr 14) were included in the pooled analysis of myocarditis among men. The pooled proportion of reported cases was 41% (95% CI: 17%–65%). Heterogeneity remained high (I² = 100%) (Figure 6).

### 3.4 Distribution of Cases by Geographic Region

**Figure 7:**
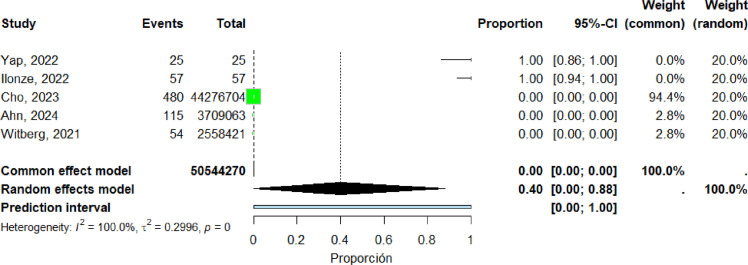
forest plot asia includes studies.

Five studies (Yap et al., 2022 Feb, Ilonze and Guglin, 2022 Nov, Cho et al., 2023 Jun 25, Ahn et al., 2024 Dec 23, Witberg et al., 2021 Dec 2) were included in the pooled analysis of myocarditis in Asia. The pooled proportion of reported cases was 40% (95% CI: 0%–88%). Heterogeneity remained high (I² = 100%) (Figure 7).

**Figure 8:**
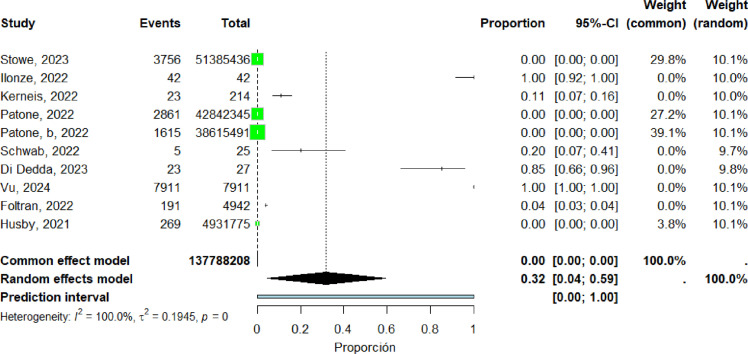
forest plot europe includes studies.

**Figure 9:**
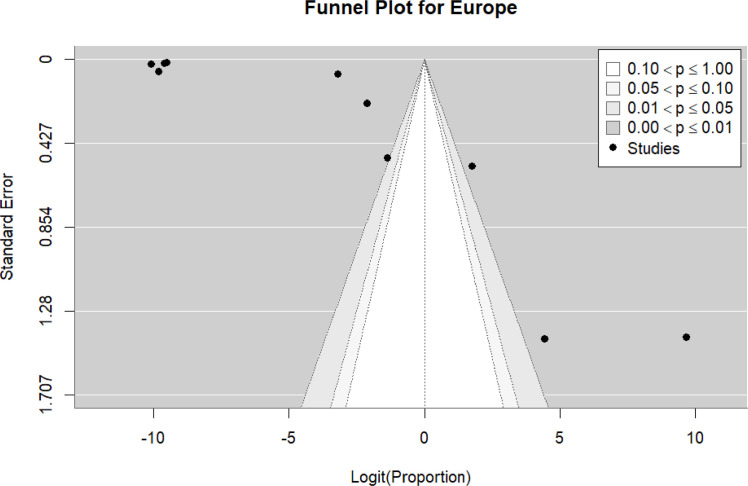
Funnel plot for the European population include studies.

Ten studies (Stowe et al., 2023 Jun 7, Ilonze and Guglin, 2022 Nov, Kerneis et al., 2021 Jun-Jul, Patone et al., 2022 Sep 6, Patone et al., 2022 Feb, Schwab et al., 2023 Mar, Di Dedda et al., 2022 Jul, Le Vu et al., 2024 Sep 5, Foltran et al., 2022 Mar 2, Husby et al., 2021 Dec 16) were included in the pooled analysis of myocarditis in Europe. The pooled proportion of reported cases was 32% (95% CI: 4%–59%). Heterogeneity remained high (I² = 100%) (Figure 8). Funnel plot inspection suggested asymmetry, indicating potential publication bias and/or small-study effects (Figure 9).

**Figure 10:**
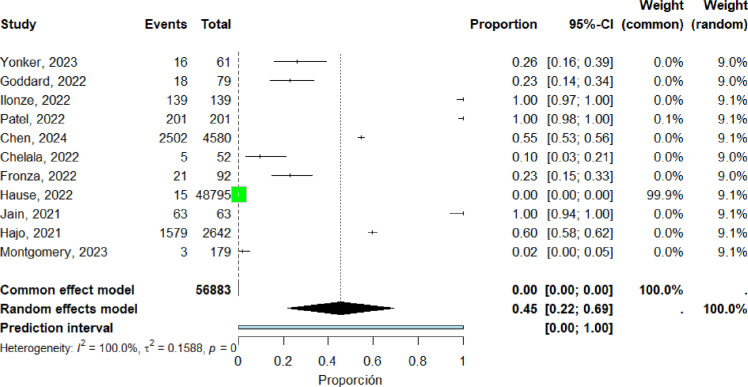
forest plot myocarditis in the Americas include studies.

**Figure 11:**
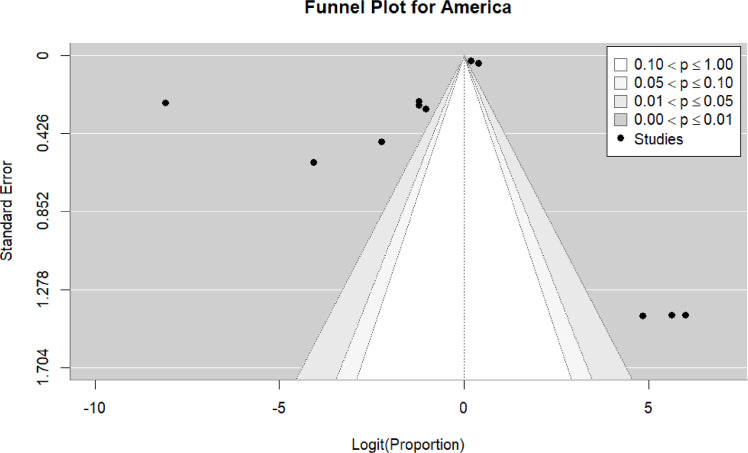
funnel plot for america include studies.

Eleven studies (Yonker et al., 2023 Mar 14, Goddard et al., 2022 Aug 19, Ilonze and Guglin, 2022 Nov, Patel et al., 2022 May 3, Chen et al., 2024 May 30, Chelala et al., 2022 Apr, Fronza et al., 2022 Sep, Hause et al., 2022 Aug 1, Jain et al., 2021 Nov, Hajjo et al., 2021 Oct 15, Montgomery et al., 2023 Jan) were included in the pooled analysis of myocarditis in the Americas. The pooled proportion of reported cases was 45% (95% CI: 22%–69%). Heterogeneity remained high (I² = 100%) (Figure 10). Funnel plot inspection suggested asymmetry, indicating potential publication bias and/or small-study effects (Figure 11).

### 3.5 Distribution of Cases by Age

**Figure 12:**
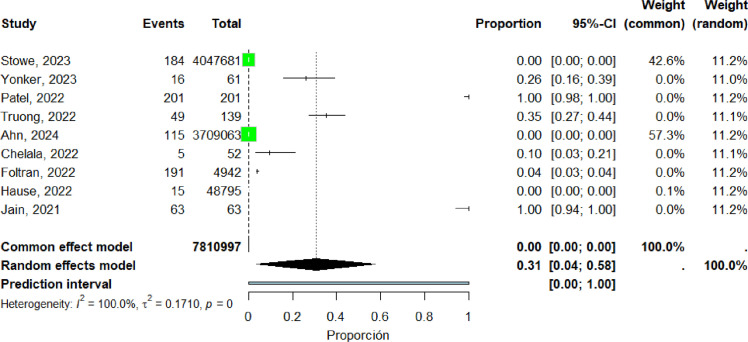
Forest plot individuals younger than 18 years include studies.

Nine studies (Stowe et al., 2023 Jun 7, Yonker et al., 2023 Mar 14, Patel et al., 2022 May 3, Truong et al., 2022 Feb, Ahn et al., 2024 Dec 23, Chelala et al., 2022 Apr, Foltran et al., 2022 Mar 2, Hause et al., 2022 Aug 1, Jain et al., 2021 Nov) were included in the pooled analysis of myocarditis among individuals younger than 18 years. The pooled proportion of reported cases was 31% (95% CI: 4%–58%). Heterogeneity remained high (I² = 100%) (Figure 12).

**Figure 13:**
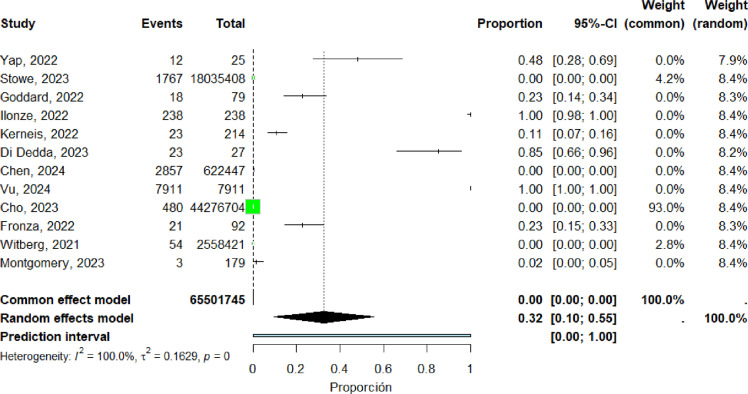
forest plot myocarditis among individuals aged 18-40 years include studies.

**Figure 14:**
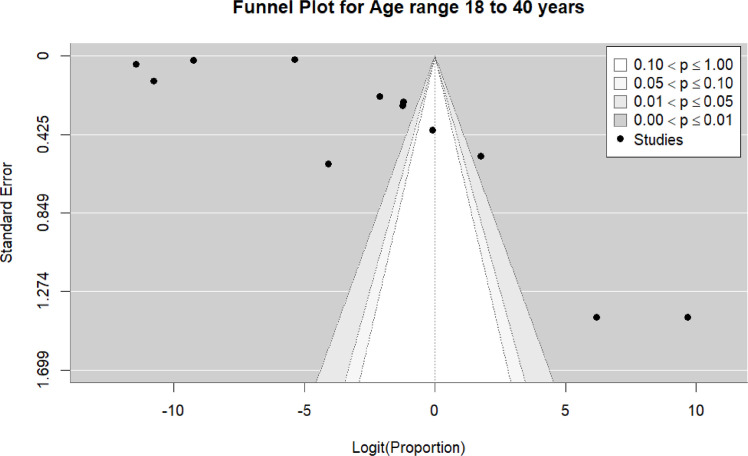
Funnel plot for the 18–40-year age subgroup includes studies.

Twelve studies (Yap et al., 2022 Feb, Stowe et al., 2023 Jun 7, Goddard et al., 2022 Aug 19, Ilonze and Guglin, 2022 Nov, Kerneis et al., 2021 Jun-Jul, Di Dedda et al., 2022 Jul, Chen et al., 2024 May 30, Le Vu et al., 2024 Sep 5, Cho et al., 2023 Jun 25, Fronza et al., 2022 Sep, Witberg et al., 2021 Dec 2, Montgomery et al., 2023 Jan) were included in the pooled analysis of myocarditis among individuals aged 18–40 years. The pooled proportion of reported cases was 32% (95% CI: 10%–55%). Heterogeneity remained high (I² = 100%) (Figure 13). Funnel plot inspection suggested asymmetry, indicating potential publication bias and/or small-study effects (Figure 14).

**Figure 15:**
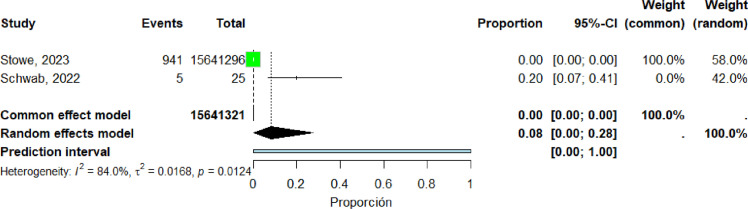
forest plot Individuals aged 41 to 60 years include studies.

Two studies (Stowe et al., 2023 Jun 7, Schwab et al., 2023 Mar) were included in the pooled analysis of myocarditis among individuals aged 41–60 years. The pooled proportion of reported cases was 8% (95% CI: 0%–28%). Heterogeneity was high (I² = 84%) (Figure 15).

### 3.6 Distribution of Cases by Vaccine Platform

**Figure 16:**
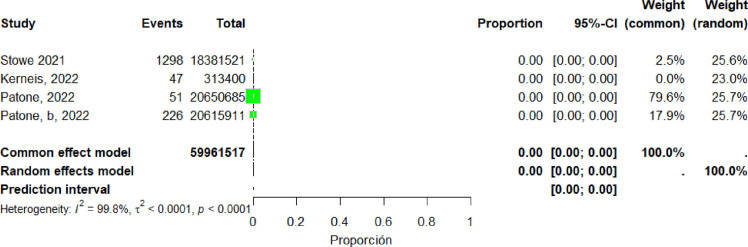
forest plot The AstraZeneca vaccine include studies.

Four studies (Stowe et al., 2023 Jun 7, Kerneis et al., 2021 Jun-Jul, Patone et al., 2022 Sep 6, Patone et al., 2022 Feb) were included in the pooled analysis of myocarditis associated with the AstraZeneca vaccine. The pooled proportion of reported cases was 0% (95% CI: 0%–0%). Heterogeneity remained high (I² = 99.9%) (Figure 16).

**Figure 17:**
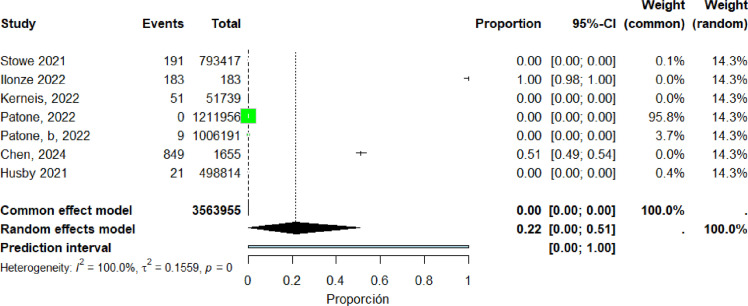
forest plot The Moderna vaccine include studies.

Seven studies (Stowe et al., 2023 Jun 7, Ilonze and Guglin, 2022 Nov, Kerneis et al., 2021 Jun-Jul, Patone et al., 2022 Sep 6, Patone et al., 2022 Feb, Chen et al., 2024 May 30, Husby et al., 2021 Dec 16) were included in the pooled analysis of myocarditis associated with the Moderna vaccine. The pooled proportion of reported cases was 22% (95% CI: 0%–51%). Heterogeneity remained high (I² = 100%) (Figure 17).

**Figure 18:**
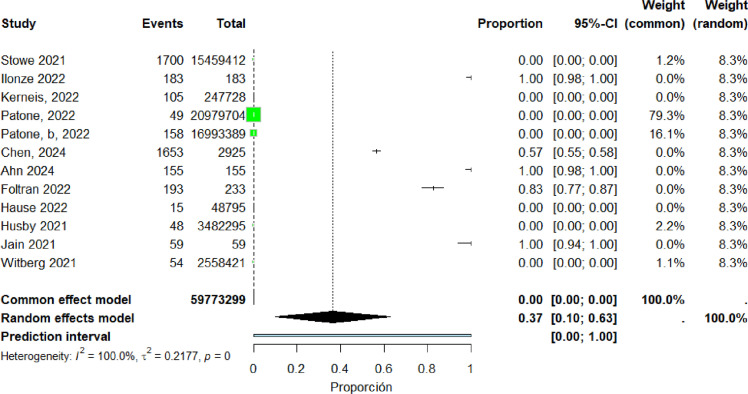
The Pfizer vaccine include studies.

**Figure 19:**
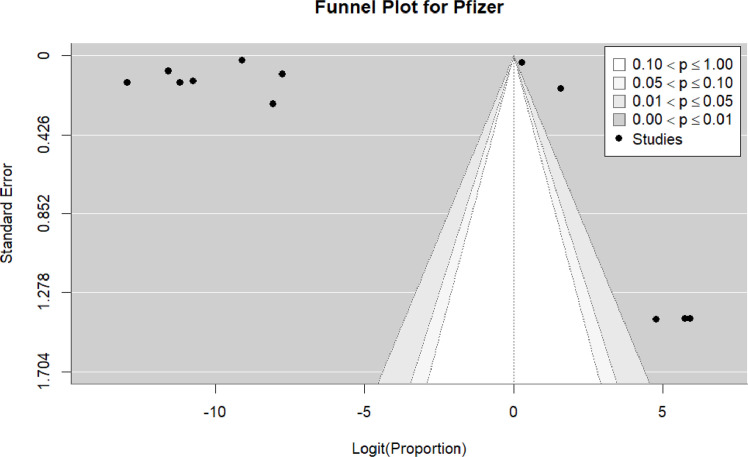
. Funnel plot for the Pfizer vaccine include studies.

Twelve studies (Stowe et al., 2023 Jun 7, Ilonze and Guglin, 2022 Nov, Kerneis et al., 2021 Jun-Jul, Patel et al., 2022 May 3, Patone et al., 2022 Sep 6, Chen et al., 2024 May 30, Ahn et al., 2024 Dec 23, Foltran et al., 2022 Mar 2, Hause et al., 2022 Aug 1, Husby et al., 2021 Dec 16, Jain et al., 2021 Nov, Witberg et al., 2021 Dec 2) were included in the pooled analysis of myocarditis associated with the Pfizer-BioNTech vaccine. The pooled proportion of reported cases was 37% (95% CI: 10%–63%). Heterogeneity remained high (I² = 100%) (Figure 18). Funnel plot inspection suggested asymmetry, indicating potential publication bias and/or small-study effects (Figure 19).

### 3.7 Distribution of Reported Deaths

**Figure 20:**
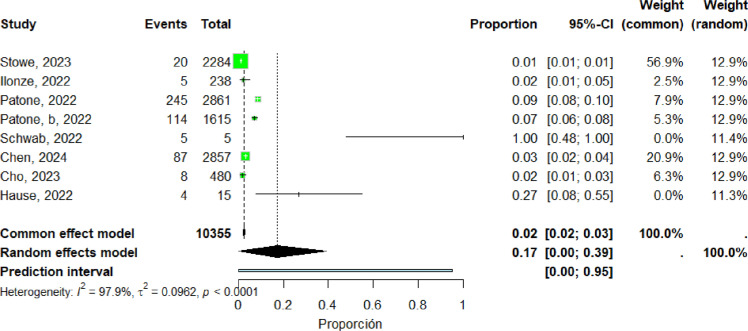
death related to post-vaccination include studies.

Eight studies (Stowe et al., 2023 Jun 7, Ilonze and Guglin, 2022 Nov, Patone et al., 2022 Sep 6, Patone et al., 2022 Feb, Schwab et al., 2023 Mar, Chen et al., 2024 May 30, Cho et al., 2023 Jun 25, Hause et al., 2022 Aug 1) were included in the pooled analysis of deaths related to post-vaccination myocarditis. The pooled proportion of reported cases was 17% (95% CI: 0%–39%). Heterogeneity remained high (I² = 97.9%) (Figure 20).

## 4 Discussion

Four previously published articles with similarities to the present study were evaluated. The first was the article by Gao et al. (2023) (Gao et al., 2023 Feb), entitled “A Systematic Review and Meta-analysis of the Association Between SARS-CoV-2 Vaccination and Myocarditis or Pericarditis.” This study addressed an objective similar to ours, namely, to determine the risk of myocarditis/pericarditis associated with COVID-19 vaccination. However, there are several notable differences. Our study analyzed age intervals up to 20 years, rather than categorizing individuals as younger or older than 40 years. In addition, we evaluated geographic distribution by continent rather than by specific countries. We also analyzed data from five different vaccine platforms (Pfizer-BioNTech, Sinovac, Sinopharm, AstraZeneca, Moderna, and Johnson & Johnson), whereas the reference article grouped vaccines into mRNA and viral vector categories. Finally, our study evaluated mortality associated with vaccine-related myocarditis, an outcome not assessed in the aforementioned report.

The second study reviewed was that by Samimisedeh et al. (2023) (Samimisedeh et al., 2024 Mar), entitled “post-acute midterm follow-up cardiac MRI findings and clinical outcomes in patients with COVID-19 vaccine-associated myocarditis: a comprehensive systematic review and meta-analysis.” Their objective differed from ours, as it focused on summarizing clinical and imaging follow-up findings in patients with vaccine-associated myocarditis. Their analysis included only individuals who developed myocarditis and emphasized post-diagnosis outcomes, whereas our study included broader populations and did not focus on longitudinal follow-up. Ishisaka et al. (2024) (Ishisaka et al., 2024 Jan 15) aimed to compare myocarditis associated with SARS-CoV-2 infection and vaccination with myocarditis unrelated to these exposures. Although they reported a higher incidence of myocarditis following infection than vaccination, their primary focus was clinical course and outcomes, including mortality and complications. Similarly, Bellos et al. (2022) (Bellos et al., 2022 Mar 15) focused on clinical characteristics and predictors of severe myocarditis, rather than on the distribution of reported cases across populations and vaccine platforms.

Overall, existing literature suggests that myocarditis following COVID-19 vaccination is an uncommon but clinically relevant adverse event (Orenstein and Rouphael, Nov 13, 2025). Several studies have reported higher frequencies of myocarditis among males and younger individuals, particularly following mRNA-based vaccines (Gao et al., 2023 Feb, Ahmed et al., 2022 Jun, Kitano et al., 2025 Jan 10). These observations are consistent with our findings, which showed a higher proportion of reported cases among males (72%) and individuals younger than 40 years. Ahmed et al. (2022) (Ahmed et al., 2022 Jun) analyzed 62 studies and found that most reported cases occurred in males and were associated with mRNA vaccines, particularly after the second dose. Clinical presentation was typically mild, with chest pain, elevated troponin, and transient cardiac dysfunction. Similarly, Samimisedeh et al. (2023) (Samimisedeh et al., 2024 Mar) reported favorable outcomes in most patients, despite persistent imaging findings such as late gadolinium enhancement in some cases. Kitano et al. (2025) (Kitano et al., 2025 Jan 10) also described higher relative frequencies of myocarditis in younger populations and after subsequent vaccine doses, although vaccination remained recommended given its overall benefits.

These findings highlight the importance of interpreting pooled proportions within the context of their methodological limitations, particularly when evaluating rare adverse events. Higher pooled proportions of reported myocarditis cases were observed among males and younger individuals, especially those aged 18–40 years. These patterns are consistent with previous studies suggesting that myocarditis following COVID-19 vaccination is more frequently reported in younger populations and after mRNA-based vaccines. However, given the substantial heterogeneity across included studies and the lack of standardized denominators, these pooled estimates should not be interpreted as measures of population-level incidence or risk. Geographic variability in reported proportions may reflect differences in surveillance systems, diagnostic criteria, reporting practices, healthcare access, and study methodology rather than true biological differences between populations. Similarly, differences observed across vaccine platforms should be interpreted cautiously because vaccine distribution, follow-up duration, reporting intensity, and study inclusion criteria varied considerably among studies. Overall, while subgroup analyses identified differences in the distribution of reported myocarditis cases, the findings primarily provide a descriptive overview of reported cases rather than comparative estimates of risk. Future studies using standardized epidemiological methods, consistent diagnostic criteria, and adequately defined comparison groups are needed to better characterize the relationship between COVID-19 vaccination and myocarditis.

The observed predominance in males has been hypothesized to be influenced by sex-related immunological differences. Estrogen has been proposed to modulate immune responses by enhancing humoral immunity and attenuating inflammatory pathways, potentially contributing to lower reported frequencies in women (Roved et al., 2017 Feb, Moulton, 2018 Oct 4). However, these mechanisms remain incompletely understood and should be interpreted cautiously. Geographic variation in reported proportions (Americas 45%, Asia 40%, Europe 32%) may reflect differences in surveillance systems, reporting practices, population characteristics, and study design rather than true differences in underlying risk. Similarly, age-related differences, with higher proportions in younger populations, may reflect more robust immune responses, whereas immunosenescence in older individuals could contribute to lower reported frequencies (Liang et al., 2022 Aug, Weiskopf and Weinberger, 2009). Among vaccine platforms, no cases were identified in the pooled analysis of studies reporting on the AstraZeneca vaccine; however, this finding should be interpreted with caution due to limited data and potential reporting bias. Comparisons across vaccine platforms should not be interpreted as evidence of differential risk, given the substantial heterogeneity in study design, denominators, and surveillance systems. Although mRNA-based vaccines have been more frequently represented in reported cases, this may reflect differences in vaccine distribution, reporting intensity, or study inclusion rather than a direct causal relationship. Several biological mechanisms have been proposed to explain myocarditis following COVID-19 vaccination. These include molecular mimicry between spike protein antigens and cardiac tissue, immune-mediated inflammatory responses, and complement activation (Sáenz-Peñas and Frías-Ordoñez, 2023 Dec, Barreiro-Pérez et al., 2023, Heidecker et al., 2022 Nov, Delves, 13 Dic 2025, Mohiddin et al., 2022, Bozkurt, 2023 Mar 14, Kadkhoda, 2022 Jan 24, Weber et al., 2014, Timofeeva et al., 2025 Sep 10). Additional hypotheses involve systemic distribution of vaccine components, anti-idiotype antibody formation, and activation of innate immune pathways. However, these mechanisms are based primarily on experimental or observational data and remain speculative. Therefore, they should not be interpreted as evidence of causality. Inadvertent intravascular administration of lipid nanoparticles has also been proposed as a potential mechanism, allowing distribution to cardiac tissue and triggering localized inflammation (Kadkhoda, 2022 Jan 24). Similarly, immune memory responses may contribute to higher reported frequencies following subsequent vaccine doses. Nevertheless, these hypotheses require further validation in well-designed prospective studies. Regarding mortality, the pooled proportion of reported deaths was 17%, although this estimate was derived from a limited number of studies and should be interpreted cautiously. Most reported cases were mild and self-limited, with favorable clinical outcomes. Importantly, the risk of myocarditis following SARS-CoV-2 infection appears to exceed that observed after vaccination in multiple studies (Ishisaka et al., 2024 Jan 15), supporting the overall benefit–risk balance of vaccination. Overall, the findings of this study should be interpreted within the context of substantial heterogeneity, variability in study design, and limitations inherent to observational data. While our results provide a descriptive overview of reported cases, they do not allow estimation of population-level risk or causal inference.

### 4.1 Limitations

A key limitation of this study is the pooling of proportions across highly heterogeneous study designs, including small case series and large-scale cohort studies. This approach does not allow estimation of true incidence or population-level risk and may lead to distorted pooled estimates. Therefore, pooled proportions should be interpreted strictly as descriptive distributions of reported cases rather than epidemiological measures of risk. Additionally, substantial statistical heterogeneity (I² = 100%) further limits the interpretability of pooled estimates and suggests the presence of underlying methodological and clinical variability across studies. Differences in case ascertainment, diagnostic criteria, and reporting systems may also have contributed to variability in observed proportions. The inclusion of data from passive surveillance systems and small case series introduces potential reporting and selection biases, which may inflate the apparent frequency of myocarditis cases in certain subgroups. Moreover, small case series inherently lack a defined denominator and are subject to substantial selection and reporting biases, whereas population-based cohort studies provide more reliable estimates of event frequency. The combination of these fundamentally different designs within a single meta-analysis likely contributed to inflated pooled proportions and limits the comparability of findings across studies. An additional methodological consideration is the potential for immortal time bias in observational studies included in this review. This bias arises when a period during which the outcome cannot occur is misclassified or excluded, potentially leading to biased interpretation of temporal associations and event frequency estimates. Given that several included studies relied on retrospective designs and administrative data sources, the presence of immortal time bias cannot be excluded. This further limits causal interpretation and highlights the need for carefully designed prospective studies to better characterize vaccine-associated myocarditis and its epidemiological patterns.

Another limitation relates to the use of the ROBINS-I tool for risk of bias assessment. While appropriate for non-randomized studies of interventions, its application to descriptive designs and case series may be less appropriate. As a result, risk of bias evaluations should be interpreted cautiously, particularly in the absence of comparator groups. Finally, the lack of consistent reporting of vaccine dose (first vs. second dose) across included studies precluded reliable dose-specific analyses, despite previously reported differences in myocarditis occurrence according to vaccine dose.

## 5 Conclusion

This systematic review and meta-analysis aimed to characterize the distribution of reported myocarditis cases following COVID-19 vaccination rather than to estimate population-level incidence or risk. Our findings describe the distribution of reported myocarditis cases following COVID-19 vaccination, particularly among younger males and after mRNA-based vaccine platforms. Although pooled proportions within reported study samples were substantial, these estimates should not be interpreted as population-level incidence or risk. The pooled proportion of myocarditis cases within reported samples was 34% (95% CI: 19%–50%), with higher proportions observed for the Pfizer-BioNTech vaccine (37%), followed by Moderna (22%). Sex-stratified analyses demonstrated substantially higher proportions among males (72%) compared with females (56%). Age-based subgroup analyses further revealed greater representation among younger individuals, particularly those under 18 years of age (31%) and those aged 18–40 years (32%). Despite these observations, existing evidence consistently indicates that the risk of myocarditis following SARS-CoV-2 infection exceeds that associated with vaccination. Moreover, vaccine-associated myocarditis is generally characterized by mild, self-limited clinical courses and favorable prognoses. Accordingly, COVID-19 vaccination remains strongly recommended as a primary preventive strategy against severe disease. Nonetheless, the identification of myocarditis as a vaccine-associated adverse event warrants continued pharmacovigilance and further well-designed epidemiological studies to better characterize underlying mechanisms and subgroups more frequently represented among reported cases while preserving the substantial public health benefits of vaccination.

**Figure:**
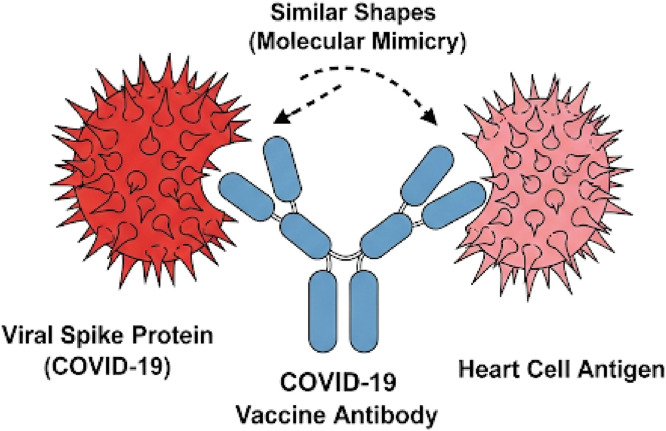
Annexes.

## CRediT authorship contribution statement

**Juan Jose Valenzuela-Fuenzalida:** Methodology, Investigation, Funding acquisition, Formal analysis, Data curation, Conceptualization. **Laura Moyano Valarezo:** Writing – original draft, Validation, Software, Project administration, Investigation, Formal analysis, Conceptualization. **Vicente Silva:** Methodology, Funding acquisition, Formal analysis, Data curation, Conceptualization. **Fernanda Delgado:** Validation, Software, Project administration, Investigation, Formal analysis, Conceptualization. **Diego Nazar-Izquierdo:** Writing – original draft, Validation, Software, Project administration, Investigation, Formal analysis, Conceptualization. **Alejandro Bruna-Mejias:** Software, Project administration, Investigation, Formal analysis, Conceptualization. **Pablo Nova-Baeza:** Writing – original draft, Validation, Software, Project administration, Investigation, Formal analysis, Conceptualization. **Mathias Orellana Donoso:** Software, Resources, Project administration, Methodology, Investigation, Funding acquisition, Formal analysis. **Gustavo Oyanedel- Amaro:** Investigation, Funding acquisition, Formal analysis, Data curation, Conceptualization. **Gloria Cifuentes-Suazo:** Supervision, Software, Resources, Investigation, Funding acquisition, Formal analysis, Conceptualization. **Maria P Moya:** Validation, Supervision, Project administration, Methodology, Investigation, Formal analysis, Conceptualization. **Juan Sanchis- Gimeno:** Project administration, Investigation, Formal analysis, Conceptualization. **Marko Konschake:** Writing – original draft, Validation, Software, Project administration, Investigation, Formal analysis, Conceptualization. **Jessica Paola Loaiza-Giraldo:** Validation, Software, Project administration, Investigation, Funding acquisition, Data curation, Conceptualization.

## Declaration of competing interest

The authors of the manuscript; “Occurrence of Myocarditis in Patients Immunized With Different Types of COVID-19 Vaccines: A Systematic Review and Meta-Analysis ” declare that they have no known competing financial interests or personal relationships that could have appeared to influence the work reported in this paper.

## Data Availability

Data will be made available on request.

## Appendix Supplementary materials

Image, application 1
